# Diagnostic imaging for chronic plantar heel pain: a systematic review and meta-analysis

**DOI:** 10.1186/1757-1146-2-32

**Published:** 2009-11-13

**Authors:** Andrew M McMillan, Karl B Landorf, Joanna T Barrett, Hylton B Menz, Adam R Bird

**Affiliations:** 1 Department of Podiatry, Faculty of Health Sciences, La Trobe University, Victoria, Australia; 2 Musculoskeletal Research Centre, Faculty of Health Sciences, La Trobe University, Victoria, Australia

## Abstract

**Background:**

Chronic plantar heel pain (CPHP) is a generalised term used to describe a range of undifferentiated conditions affecting the plantar heel. Plantar fasciitis is reported as the most common cause and the terms are frequently used interchangeably in the literature. Diagnostic imaging has been used by many researchers and practitioners to investigate the involvement of specific anatomical structures in CPHP. These observations help to explain the underlying pathology of the disorder, and are of benefit in forming an accurate diagnosis and targeted treatment plan. The purpose of this systematic review was to investigate the diagnostic imaging features associated with CPHP, and evaluate study findings by meta-analysis where appropriate.

**Methods:**

Bibliographic databases including Medline, Embase, CINAHL, SportDiscus and The Cochrane Library were searched electronically on March 25, 2009. Eligible articles were required to report imaging findings in participants with CPHP unrelated to inflammatory arthritis, and to compare these findings with a control group. Methodological quality was evaluated by use of the Quality Index as described by Downs and Black. Meta-analysis of study data was conducted where appropriate.

**Results:**

Plantar fascia thickness as measured by ultrasonography was the most widely reported imaging feature. Meta-analysis revealed that the plantar fascia of CPHP participants was 2.16 mm thicker than control participants (95% CI = 1.60 to 2.71 mm, *P *< 0.001) and that CPHP participants were more likely to have plantar fascia thickness values greater than 4.0 mm (OR = 105.11, 95% CI = 3.09 to 3577.28, *P *= 0.01). CPHP participants were also more likely to show radiographic evidence of subcalcaneal spur than control participants (OR = 8.52, 95% CI = 4.08 to 17.77, *P *< 0.001).

**Conclusion:**

This systematic review has identified 23 studies investigating the diagnostic imaging appearance of the plantar fascia and inferior calcaneum in people with CPHP. Analysis of these studies found that people with CPHP are likely to have a thickened plantar fascia with associated fluid collection, and that thickness values >4.0 mm are diagnostic of plantar fasciitis. Additionally, subcalcaneal spur formation is strongly associated with pain beneath the heel.

## Background

Chronic plantar heel pain (CPHP) is a generalised term used to describe a range of undifferentiated conditions affecting the plantar heel. Clinical features are typically described as chronic pain beneath the heel, made worse by weight-bearing after prolonged periods of rest [1]. Plantar fasciitis is reported as the most common cause of CPHP [2] and the terms are frequently used interchangeably in the literature [3]. CPHP is also associated with inflammatory conditions such as spondyloarthritis [4], though the majority of cases are unrelated to systemic disease [5].

The epidemiology of CPHP in the general population is currently uncertain. An Australian population-based study involving 3,206 randomly selected participants has reported a heel pain prevalence of 3.6% [6]. American studies estimate that 7% of older adults report tenderness beneath the heel [7], and that 1 million physician consultations per year are for the diagnosis and treatment of plantar fasciitis [8]. Plantar fasciitis is also estimated to account for approximately 8% of all running-related injuries [9,10].

Diagnostic imaging has been used by many researchers and practitioners to investigate the involvement of specific anatomical structures in CPHP. Imaging types used include ultrasonography and magnetic resonance imaging (MRI) for investigation of soft tissue structures (e.g. the plantar fascia) and plain film x-rays for bone abnormalities (e.g. heel spur). These observations help to explain the underlying pathology of the disorder, and are of benefit in forming an accurate diagnosis and targeted treatment plan. Additionally, these studies provide objective criteria by which to measure the effect of current and future treatments.

At the time of writing, one published article had attempted to critically review diagnostic imaging studies for plantar fasciitis [11]. However, this review had a broad scope including both assessment and treatment studies, and did not include all available diagnostic imaging research. Furthermore, the article presented only a limited overview of study findings and did not investigate other potential causes of CPHP (e.g. heel spur). Therefore, the objective of this systematic review was to investigate all diagnostic imaging features associated with CPHP and evaluate study findings by meta-analysis where appropriate.

## Methods

### Search strategy and eligibility criteria

A systematic review was conducted using the following bibliographic databases: Medline, Embase, CINAHL, SportDiscus and The Cochrane Library. Databases were searched electronically on March 25, 2009 and 'auto-alerts' were designed to deliver weekly updates of additional citations until June 30, 2009. A detailed description of the search strategy is available in Additional File 1.

Studies included in the review were required to be published in peer-reviewed journals and to describe original research findings in the English language. Included studies had to report diagnostic imaging findings in participants with CPHP and compare these findings with an independent control group. For the purpose of this review, CPHP was defined as chronic pain localised beneath the heel, made worse by weight-bearing after prolonged periods of rest [1]. This definition was used to encompass a variety of clinical diagnostic terms such as plantar fasciitis, plantar heel pain and heel spur syndrome. Studies included in this review were required to either describe the signs and symptoms of participants as being consistent with this definition, or to state a diagnosis known primarily by these clinical features (e.g. clinical diagnosis of plantar fasciitis).

Studies in which comparisons were exclusively made between the symptomatic and asymptomatic feet of participants with unilateral CPHP were excluded. This decision was based on evidence that the asymptomatic foot of people with unilateral CPHP may demonstrate osseus [12] and soft tissue [13,14] abnormalities when compared to people without CPHP. Studies exclusively investigating disease-specific cohorts (such as autoimmune disease and diabetes mellitus), neurovascular abnormalities, plantar fibromatosis and biomechanical variables were also excluded. Plantar heel pad investigations were excluded because they were considered to relate more to risk factors associated with CPHP rather than the identification of underlying pathology. All citations generated by the search strategy were examined by two assessors according to the criteria described above.

### Assessment of methodological quality and diversity

Methodological quality was assessed by two authors (AM and JB) who were blinded to author and publication details. The assessment tool used for this process was a modified version of the Quality Index originally described by Downs and Black [15]. A detailed description of the quality assessment tool is available in Additional File 2.

Outcome data and information regarding the overall study design, subject characteristics and imaging techniques were obtained by two authors (AM and JB) with use of a standardised data extraction form. Studies were grouped according to commonly reported imaging features (e.g. plantar fascia thickness) and then by imaging modality (e.g. ultrasonography). The clinical and methodological diversity between studies was assessed to determine the appropriateness of pooling data for meta-analysis. Factors considered important for comparison included the mean age, sex distribution, mean BMI and comorbidity of both condition and control groups. The clinical characteristics of the condition group, technical imaging equipment used and outcome measurement techniques were also compared. Two authors (AM and KL) compared studies according to these features and reached consensus on the appropriateness of progressing to meta-analysis.

### Data analysis

All data analyses were performed by use of Review Manager software (RevMan Version 5.0.14. Copenhagen: The Nordic Cochrane Centre, The Cochrane Collaboration, 2008). Statistical heterogeneity between studies was assessed by use of I^2 ^and Chi^2 ^statistics. Values of I^2 ^range between 0% and 100% and describe the percentage of variability across study findings that is due to heterogeneity rather than chance alone [16]. Heterogeneity was considered low if the I^2 ^value was 25% or less, moderate if the value was between 25% and 50%, high if between 50% and 75% and very high if greater than 75% [17]. Chi^2 ^was performed with *P *< 0.1 considered statistically significant due to the low power of this test in detecting heterogeneity (i.e. to increase the chance of detecting heterogeneity a higher *P *value was chosen) [16]. Meta-analysis occurred by the fixed-effect method where the I^2 ^statistic was less than 50% and the Chi^2 ^test indicated a non-significant degree of heterogeneity (*P *> 0.1). The random-effect method was used where the I^2 ^statistic was greater than 50% and the Chi^2 ^test indicated statistically significant heterogeneity (*P *< 0.1). Meta-analysis by the random-effect method incorporates heterogeneity into the analysis, resulting in a wider confidence interval and a more conservative claim of statistical significance [16].

Continuous data were analysed by obtaining the mean values, standard deviations (SD) and sample size for each study within the group. The difference in means and 95% confidence interval (CI) for each individual study were calculated, and the weighted pooled estimate determined by the inverse-variance method. For dichotomous data, the odds ratio (OR) and 95% CI for each individual study were calculated, and the weighted pooled estimate determined by the inverse variance method. Sensitivity analysis was performed to exclude studies that did not apply a blinding technique to the image assessor. Sub-group analysis was performed for groups containing ten studies or more [16] to compare the pooled estimates of blinded and non-blinded studies. However, formal sub-group comparisons were not performed for heterogeneous data (I^2 ^> 50%, *P *< 0.1) to reduce the risk of false positive results [16].

Bias within groups containing 10 studies or more was assessed by use of a funnel plot, in which effect estimates of individual studies are plotted on the horizontal axis against their standard error on the vertical axis [18]. In the absence of bias, effect estimates of smaller studies are scattered at the lower end of the plot with larger studies clustered centrally towards the top, thereby forming a symmetrical inverted funnel [19]. Absence of effect estimates in the lower corners of funnel plots were interpreted as evidence of bias, suggesting the difference between groups may be overestimated by meta-analysis [18].

Eligibility criteria, quality assessment procedures and methods of data analysis were specified prospectively and outlined in an unpublished review protocol.

## Results

A total 764 citations were identified by the database search process (Additional file 3), of which 23 studies were included in the review (Additional file 4). The exclusion grounds for articles rejected after full-text assessment are available in Additional file 5.

Quality index scores ranged from 29 to 80% (mean = 55%) demonstrating moderate overall methodological quality (Additional file 6). The majority of studies provided inadequate descriptions of control group source populations and characteristics, and only eight studies [12,13,20-25] applied a blinding technique to the image assessor. Additionally, 11 studies [12,13,22-24,26-31] included data from both feet of control participants, and 11 studies [12,13,21,22,24,26-29,31,32] included data from both feet of participants with bilateral heel pain. As statistical tests assume that each data point represents a truly independent observation, inclusion of both feet may result in an artificially inflated sample size and decreased data variability, thereby increasing the risk of Type I error [33]. Despite these limitations, most studies reported predetermined outcome variables, and clearly described imaging equipment settings and measurement techniques.

### Thickness of the proximal plantar fascia

The thickness of the proximal plantar fascia was reported in 15 studies, 12 of which were measured by ultrasonography alone [13,14,20,22,25,28-31,34-36], one by ultrasonography and magnetic resonance imaging (MRI) [23], one by MRI alone [27], and one by plain film x-ray [21]. A factor considered important for this outcome was the prevalence of diabetes mellitus within each group, as research has shown a thickening of the plantar fascia in people with diabetes [37]. Only two studies [21,22] considered diabetes as a specific exclusion criterion for condition groups, and only one study [22] for the control group.

#### Ultrasonography

The 13 studies reporting plantar fascia thickness by ultrasonography had a mean quality index score of 56%. Five studies [13,20,22,23,25] applied a blinding technique to the image assessor. A description of the methodological variability between studies is available in Additional file 7.

As the protocols and participant characteristics of the studies reporting this outcome were found to be similar, meta-analysis was considered appropriate. However, two studies could not be included: one study [31] reported data separately for the medial, central and lateral components of the plantar fascia, and another study [30] did not report the standard deviation of the mean plantar fascia thickness values.

**Figure 1:**
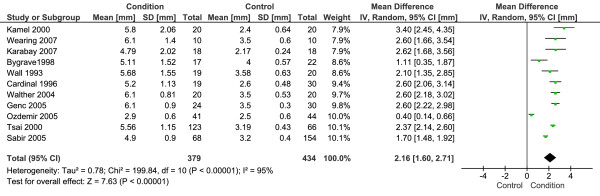
**Forest plot of studies reporting the thickness of the proximal plantar fascia by ultrasonography**.

**Figure 2:**
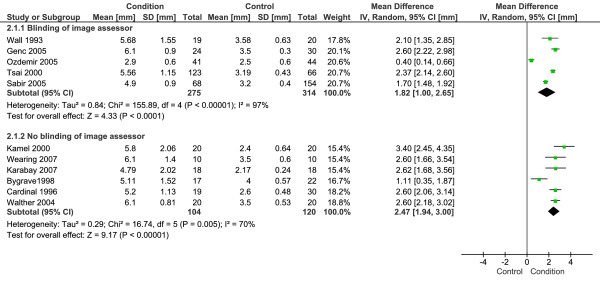
**Forest plot of studies reporting the thickness of the proximal plantar fascia by ultrasonography**. Sub-group analysis: blinding versus no blinding of image assessor.

**Figure 3:**
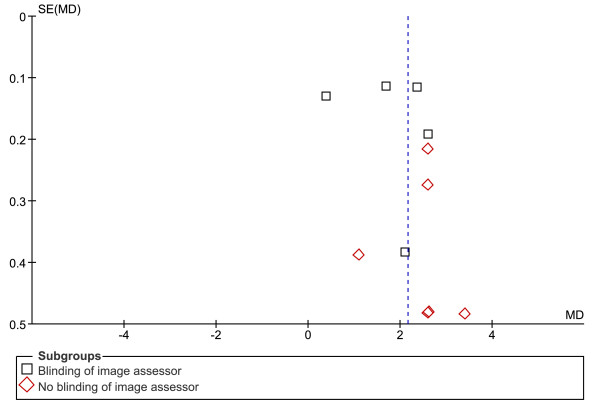
**Funnel plot of studies reporting the thickness of the proximal plantar fascia by ultrasonography**. Sub-group analysis: blinding versus no blinding of image assessor.

Eleven studies with a total 379 CPHP participants and 434 control participants were included in this analysis. Statistical heterogeneity between studies was very high (I^2 ^= 95%; Chi^2 ^= 199.84, df = 10, *P *< 0.001), therefore meta-analysis was undertaken using the random-effect method. The mean difference between groups was statistically significant (*P *< 0.001), with the proximal plantar fascia of CPHP participants 2.16 mm thicker than control participants (95% CI = 1.60 to 2.71 mm) (Figure 1). Sub-group analysis revealed a more conservative pooled estimate by studies that applied a blinding technique to the image assessor. The mean difference between groups for blinded studies was 1.82 mm (95% CI = 1.00 to 2.65 mm, *P *< 0.001) and for non-blinded studies was 2.47 mm (95% CI = 1.94 to 3.00 mm, *P *< 0.001) (Figure 2). Funnel plot inspection revealed that studies were absent from the lower left corner of the plot, suggesting that smaller studies reporting less difference between groups had not been published. However, this distribution was explained by identifying studies on the plot that applied a blinding technique to the image assessor (Figure 3). Non-blinded studies had smaller sample sizes than the majority of blinded studies, and as a result appeared lower on the plot. Therefore, the funnel plot distribution illustrates that non-blinded studies reported larger mean differences between groups than the majority of blinded studies, indicating an overestimation of the thickness of the plantar fascia in CPHP groups.

**Figure 4:**
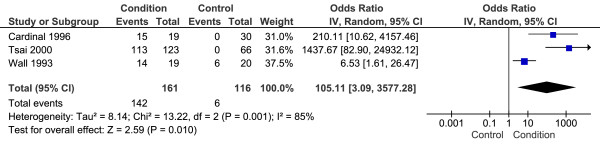
**Forest plot of ultrasonography studies reporting proximal plantar fascia thickness values > 4.0 mm**.

Two studies included in the analysis above [13,29] also reported the proportion of participants in each group with plantar fascia thickness values > 4.0 mm (i.e. the thickness values for participants were dichotomised). Additionally, one study included in the analysis above [25] reported the individual thickness values for each participant, allowing dichotomisation for the purpose of this review. These studies included a total 161 CPHP participants and 116 control participants. Statistical heterogeneity between studies was very high (I^2 ^= 85%; Chi^2 ^= 13.22, df = 2, *P *= 0.001), therefore meta-analysis was undertaken using the random-effect method. The mean difference between groups was statistically significant (*P *= 0.01) with CPHP participants over 100 times more likely than control participants to have plantar fascia thickness values > 4.0 mm (OR = 105.11, 95% CI = 3.09 to 3577.28) (Figure 4).

#### MRI

Two studies measured the thickness of the proximal plantar fascia by MRI. One study [23] applied a blinding technique to the image assessor and had a quality index score of 70%, the other [27] did not blind the image assessor and had a quality index score of 43%. A description of the methodological variability between studies is available in Additional file 8. As the protocols and participant characteristics of the studies reporting this outcome were found to be similar, meta-analysis was considered appropriate.

**Figure 5:**
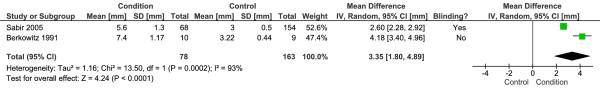
**Forest plot of studies reporting the thickness of the proximal plantar fascia by MRI**.

Two studies with a total 78 CPHP participants and 163 control participants were included in this analysis. Statistical heterogeneity between studies was very high (I^2 ^= 93%; Chi^2 ^= 13.50, df = 1, *P *< 0.001), therefore meta-analysis was undertaken using the random-effect method. The mean difference between groups was statistically significant (*P *< 0.001), with the proximal plantar fascia of CPHP participants 3.35 mm thicker than control participants (95% CI = 1.80 to 4.89 mm) (Figure 5). The blinded study reported a more conservative difference between groups (2.60 mm, 95% CI = 2.28 to 2.92 mm) than the non-blinded study (4.18 mm, 95% CI = 3.40 to 4.96 mm).

#### Plain film x-ray

One study [21] measured the thickness of the proximal plantar fascia by plain film x-ray. This study had a quality index score of 68% and applied a blinding technique to the image assessor. The sagittal thickness of the plantar fascia was measured from a lateral non-weight bearing radiograph within 5.0 mm of the calcaneal insertion. This study reports a statistically significant mean difference between groups, with the plantar fascia of CPHP participants 2.4 mm thicker than control participants (*P *< 0.001). The 95% CI for the difference between groups was not reported.

### Ultrasound echogenicity and MRI signal intensity of the proximal plantar fascia

Four studies reported the echogenicity (presence or absence of fluid collection) of the proximal plantar fascia [13,20,29,36]. The mean quality index score was 60%, and two studies [13,20] applied a blinding technique to the image assessor. A description of the methodological variability between studies is available in Additional file 9. As the protocols and participant characteristics of the studies reporting this outcome were found to be similar, meta-analysis was considered appropriate.

**Figure 6:**
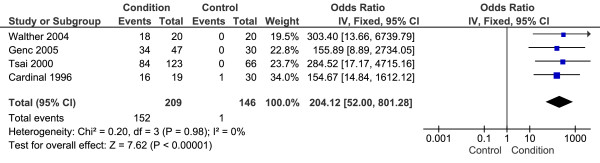
**Forest plot of studies reporting hypoechogenicity of the proximal plantar fascia**.

Four studies with a total 209 CPHP participants and 146 control participants were included in this analysis. Statistical heterogeneity between studies was low (I^2 ^= 0%; Chi^2 ^= 0.20, df = 3, *P *= 0.98), therefore meta-analysis was undertaken using the fixed-effect method. The mean difference between groups was statistically significant (*P *< 0.001) with CPHP participants over 200 times more likely to demonstrate hypoechogenicity of the proximal plantar fascia than control participants (OR = 204.12, 95% CI = 52.00 to 801.28) (Figure 6). Sensitivity analysis revealed an increased pooled estimate after exclusion of the two [29,36] non-blinded studies (OR = 211.87, 95% CI = 28.53 to 1573.54, *P *< 0.001).

One study [27] reported the MRI signal intensity (presence or absence of fluid collection) of the proximal plantar fascia. This study had a quality index score of 43% and did not apply a blinding technique to the image assessor. Increased signal intensity was observed in the region of fascia thickening for CPHP participants, compared with homogenous low signal intensity of the plantar fascia in all control participants.

### Evidence of plantar calcaneal spur

Seven studies reported evidence of plantar calcaneal spur by plain film x-ray [12,21,24,26,38-40]. The mean quality index score was 58% and only three studies [12,21,24] applied a blinding technique to the image assessor. A description of the methodological variability between studies is available in Additional file 10. As the protocols and participant characteristics of the studies reporting this outcome were found to be similar, meta-analysis was considered appropriate.

**Figure 7:**
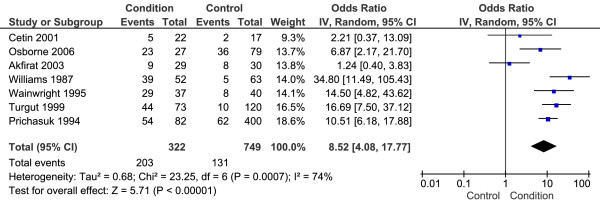
**Forest plot of studies reporting evidence of plantar calcaneal spur by plain film x-ray**.

Seven studies with a total 322 CPHP participants and 749 control participants were included in this analysis. Statistical heterogeneity between studies was high (I^2 ^= 74%; Chi^2 ^= 23.25, df = 6, *P *< 0.001), therefore meta-analysis was undertaken using the random-effect method. The mean difference between groups was statistically significant (*P *< 0.001) with CPHP participants over 8 times more likely to show evidence of subcalcaneal spur than control participants (OR = 8.52, 95% CI = 4.08 to 17.77) (Figure 7). Sensitivity analysis revealed an increased pooled estimate after exclusion of the four [26,38-40] non-blinded studies (OR = 16.11, 95% CI = 7.09 to 36.60, *P *< 0.001).

One study [30] reported evidence of subcalcaneal spur by ultrasonography. This study had a quality index score of 43% and did not apply a blinding technique to the image assessor. A variable frequency (5-10 MHz) linear array transducer was used to assess the heels of 190 CPHP and 48 control participants. The presence of subcalcaneal spur was a subjective observation found in 45% of CPHP participants and only 2% of control participants.

### Radioisotope uptake and vascular perfusion of the proximal plantar fascia

**Figure 8:**
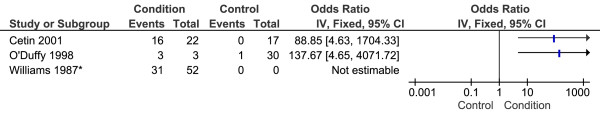
**Forest plot of studies reporting increased subcalcaneal radioisotope uptake**. *Control group sample size not reported.

Three studies reported the presence of increased radioisotope uptake within the subcalcaneal region in participants with CPHP [12,40,41]. The mean quality index score was 45% and one study [12] applied a blinding technique to the image assessor. Meta-analysis of data from these studies was not found to be appropriate as one study did not report the control group sample size [12], and another did not report the phase (early or delayed) in which scintigraphic images were assessed [40]. Participant characteristics of condition [41] and control [12] groups were also poorly reported. All three studies reported increased subcalcaneal uptake of technetium-99 m methylene diphosphonate in participants with CPHP compared to control groups (Figure 8), though no statistical comparisons were made.

One study described the degree of vascular perfusion within the proximal plantar fascia by use of power doppler ultrasound [36]. This study had a quality index score of 47% and did not apply a blinding technique to the image assessor. Doppler ultrasound with a pulse-repetition frequency of 1102 Hz was used to grade the colour signal of the proximal plantar fascia. This study reported moderate to marked hyperaemia of the proximal plantar fascia in 8 of 20 CPHP participants, and only mild hyperaemia in 1 of 20 control participants. The difference between groups was not statistically analysed, however the authors report a statistically significant correlation between hyperaemia and symptom duration of less than six months (Spearman *r *= -0.68, *P *< 0.05).

## Discussion

The objective of this systematic review was to investigate the diagnostic imaging features associated with CPHP and evaluate study findings by meta-analysis where appropriate. The majority of studies provided inadequate descriptions of control group characteristics and only a small proportion applied a blinding technique to the image observer. Furthermore, by including data from both feet of participants, the sample sizes of approximately half the studies were inappropriately inflated. While the clinical signs and symptoms of CPHP participants were similarly described across the studies, various diagnostic terms have been used, including plantar fasciitis, painful heel syndrome and inferior calcaneal spur syndrome.

The studies included in this review describe a fusiform thickening of the plantar fascia close to the calcaneal enthesis, with associated fluid collection and increased vascularity. These findings suggest that many patients with chronic pain beneath the heel are likely to have plantar fasciitis, and that changes in the thickness of the plantar fascia may be particularly useful in diagnosing the condition. For example, plantar fascia thickness values greater than 4.0 mm have previously been used to form a case definition in plantar fasciitis research [23]. This reference value is supported by the data analysis of this review, though the threshold value of 4.0 mm relates only to measurement by ultrasonography.

Plantar fascia thickness values have also been used to measure the effect of treatments. For example, corticosteroid injection has been shown to significantly reduce plantar fascia thickness as early as two weeks [42] and one month [20] following treatment. Additionally, one of these studies [20] reports a statistically significant correlation between decreased plantar fascia thickness and improvement in symptoms (Pearson *r *= 0.61, *P *< 0.001). The intra-rater reliability of measuring plantar fascia thickness by ultrasonography has been reported to be very good, with the 95% limits of agreement ranging from -0.7 mm to 0.5 mm [43]. However, the reliability of this technique has not been examined in detail.

In addition to fascia thickening, areas of hypoechogenicity within the proximal plantar fascia have also been commonly reported and are strongly associated with CPHP. Sonographic studies have attributed this feature to the presence of underlying reparative processes, with associated fibre deterioration and tissue oedema [29,36]. Evidence from histopathological studies in plantar fasciitis support this view, with increased mucoid ground substance, collagen degeneration and angiofibroblastic hyperplasia the most commonly reported features [44]. However, markers of persistent inflammation such as lymphocyte and macrophage infiltration have been less frequently reported in the condition [44]. This suggests that plantar fasciitis may follow a similar pathological pathway to that of tendinopathy, where tissue changes are thought to proceed from an early reactive phase to progressive degeneration [44,45]. Imaging studies in tendinopathy provide evidence of similarity between these conditions, in which tendon appears thickened with focal areas of hypoechogenicity and increased vascularity [45,46]. Furthermore, tendon has been found to respond to corticosteroid injection in a similar way to the plantar fascia, with one study reporting a significant reduction in tendon diameter as early as one week following treatment [47]. Future longitudinal research investigating the imaging features and histology of plantar fasciits would be of great value, as direct evidence for pathological change over time is currently lacking. This concept has particular relevance to the management of CPHP, as future interventions may be selected according to condition chronicity.

The role of subcalcaneal spur in the pathogenesis of CPHP has been questioned in musculoskeletal medicine for several decades [39,44]. The basis of this uncertainty is the reportedly high prevalence of subcalcaneal spur in the asymptomatic population [38], leading to an emerging view that the finding has limited diagnostic value [2]. However, comparisons to asymptomatic control groups in the statistical analysis of this review (odds ratio) demonstrate a strong association between CPHP and the presence of subcalcaneal spur. Inconsistencies in the association between spur formation and heel pain have not been adequately investigated, but possible explanations include variations in spur length (i.e. longer spurs may be more symptomatic) [39] and concurrent fat pad abnormalities [21,24].

As the majority of studies investigating the presence of subcalcaneal spur used plain film x-ray, the precise relationships between spur formation and surrounding soft tissue were not reported. Nonetheless, one study described the location of spurs as being closely associated with the abductor hallucis and flexor digitorum brevis origins [21]. This finding is consistent with evidence from cadaveric research, in which subcalcaneal spurs are reported to most commonly occur immediately deep to the plantar fascia enthesis [48].

The formation of subcalcaneal spur has traditionally been attributed to repetitive longitudinal traction of the plantar fascia [39], with subsequent inflammation and reactive ossification [49]. However, recent histological and clinical studies suggest that vertical compressive forces may play a more important role [48,49]. Histological evidence shows that: spur formation can occur in loose connective tissue, surrounding fibrocartilage may not be aligned with the direction of traction, and spur trabeculae commonly forms perpendicular to its long axis [48]. Additionally, clinical studies have shown that spur development is unrelated to medial arch height [49] and can occur after surgical release of the plantar fascia [50].

In the clinical management of CPHP, diagnostic imaging can provide objective information by which to either confirm or question the diagnosis of plantar fasciitis. This information can be particularly useful in cases that do not respond to first-line interventions, or when considering more invasive treatments (e.g. corticosteroid injection). The presence of a subcalcaneal spur in patients with CPHP is also likely to be an important finding, though a causal relationship has not been established. Further research involving the use of MRI and histological techniques is required to better define the role of spur formation and related bony abnormalities in the development of CPHP.

While this review was designed to be as comprehensive as possible, it is feasible that some studies that may have been suitable were not identified. In addition, as this review only included studies in which comparisons were made to asymptomatic control groups, many case-series studies and individual case reports have not been included. Therefore, the findings of this review are not exhaustive and do not describe all imaging features associated with CPHP.

## Conclusion

This systematic review has identified 23 studies investigating the diagnostic imaging appearance of the plantar fascia and inferior calcaneum in people with CPHP. Analysis of these studies found that people with CPHP are likely to have a thickened plantar fascia with associated fluid collection, and that thickness values >4.0 mm are diagnostic of plantar fasciitis. Additionally, subcalcaneal spur formation is strongly associated with pain beneath the heel.

## Competing interests

HBM and KBL are Editor-in-Chief and Deputy Editor-in-Chief, respectively, of the *Journal of Foot and Ankle Research*. It is journal policy that editors are removed from the peer review and editorial decision making processes for papers they have co-authored.

## Authors' contributions

AMM led and designed the review, carried out searches and eligibility checks, extracted study data and performed the quality assessment, evaluated the appropriateness of pooling data, performed the meta-analyses, interpreted the findings and drafted the manuscript. KBL assisted in designing the review, evaluated the appropriateness of pooling data, assisted in the interpretation of findings and commented on the draft manuscript. JTB extracted study data, performed the quality assessment and commented on the draft manuscript. HBM assisted in the interpretation of findings and commented on the draft manuscript. ARB assisted in the review process and commented on the draft manuscript.

All authors read and approved the final manuscript.

## Supplementary Material

Additional file 1 **Description of search strategy**. A detailed description of the database search strategy. Click here for file

Additional file 2 **Description of quality assessment tool**. A detailed description of the Downs and Black quality assessment tool. Click here for file

Additional file 3 **Search results by database**. A table showing the number of citations generated by the search strategy for each database. Click here for file

Additional file 4 **Included studies**. A table showing the author and publication details of the included studies. Click here for file

Additional file 5 **Exclusion grounds for articles rejected after full-text assessment**. A table showing the exclusion grounds for articles excluded from the review after full-text assessment. Click here for file

Additional file 6 **Quality Index Scores**. A table showing the individual quality index scores for each included article. Click here for file

Additional file 7 **Thickness of the proximal plantar fascia by ultrasonography: variability between studies**. A detailed description of the methodological variability between studies reporting plantar fascia thickness by ultrasonography. Click here for file

Additional file 8 **Thickness of the plantar fascia by MRI: variability between studies**. A detailed description of the methodological variability between studies reporting plantar fascia thickness by MRI. Click here for file

Additional file 9 **Echogenicity of the proximal plantar fascia: variability between studies**. A detailed description of the methodological variability between studies reporting plantar fascia echogenicity. Click here for file

Additional file 10 **Evidence of plantar calcaneal spur by plain film x-ray: variability between studies**. A detailed description of the methodological variability between studies reporting evidence of plantar calcaneal spur by plain film x-ray. Click here for file
