# Supplementary material for: Diagnostic imaging for chronic plantar heel pain: a systematic review and meta-analysis
Source: J Foot Ankle Res. 2009 Nov 13;2:32. doi: 10.1186/1757-1146-2-32 (PMC2784446; doi:10.1186/1757-1146-2-32)
Supplement: Additional file 1 — Description of search strategy. A detailed description of the database search strategy. [file 1757-1146-2-32-S1.pdf]

Diagnostic imaging for chronic plantar heel pain: a systematic review and meta-analysis

Andrew M. McMillan, Karl B. Landorf, Joanna T. Barrett, Hylton B. Menz, Adam R. Bird

Additional Data File 1. Description of search strategy.

Database-specific subject terms and truncated keywords were initially sorted into two distinct concepts: (a) chronic plantar heel pain and (b) diagnostic imaging. Within each concept, subject terms were searched individually while keywords were combined with the term 'OR' into a single search. Subject terms and keywords within each concept were then combined with the term 'OR' to remove duplicates. Following this, the final search results for the two concepts were combined together with the term 'AND' to form the total yield. This technique was applied to Medline (Supplementary Table 1), Embase and CINAHL. SportDiscus and the Cochrane Library were searched by use of keywords only, as subject term functions were not available in these databases. Targeted searching of relevant journals also occurred following bibliographic review of retrieved articles.

Supplementary Table 1. Medline search strategy

**a**	1	Subject Term	exp. Fasciitis, Plantar
	2	Subject Term	exp. Heel Spur
	3	Keywords	Plantar Fasci* [or] Calcaneal Enthes* [or] Calcaneal Periostitis [or] Calcaneodynia [or] Epin Calcanei [or] Heel Enthes* [or] Heel Pain [or] Heel Syndrome [or] Painful Heel* [or] Subcalcaneal [or] Sub-calcaneal [or] Spur
**b**	4	Subject Term	exp. Pathology
	5	Subject Term	exp. Diagnostic Imaging
	6	Subject Term	exp. Magnetic Resonance Imaging
	7	Subject Term	exp. Ultrasonography
	8	Subject Term	exp. Radiography
	9	Subject Term	exp. Tomography
	10	Subject Term	exp. Radionuclide Imaging
	11	Keywords	Patholog* [or] Image* [or] Imaging [or] Scan* [or] MR* [or] Ultrasonograph* [or] Sonograph* [or] Echograph* [or] Ultrasound [or] US [or] Doppler [or] Radio* [or] X-Ray* [or] X Ray* [or] XRay* [or] Tomograph* [or] CT [or] Scinti*
**c**	12	Combine	1 [or] 2 [or] 3
	13	Combine	4 [or] 5 [or] 6 [or] 7 [or] 8 [or] 9 [or] 10 [or] 11
	14	Combine	12 [and] 13

(a) Search terms for chronic plantar heel pain (b) Search terms for diagnostic imaging (c) Combination of search terms.
