# Supplementary material for: Diagnostic imaging for chronic plantar heel pain: a systematic review and meta-analysis
Source: J Foot Ankle Res. 2009 Nov 13;2:32. doi: 10.1186/1757-1146-2-32 (PMC2784446; doi:10.1186/1757-1146-2-32)
Supplement: Additional file 2 — Description of quality assessment tool. A detailed description of the Downs and Black quality assessment tool. [file 1757-1146-2-32-S2.pdf]

Diagnostic imaging for chronic plantar heel pain: a systematic review and meta-analysis

Andrew M. McMillan, Karl B. Landorf, Joanna T. Barrett, Hylton B. Menz, Adam R. Bird

Additional Data File 2. Description of quality assessment tool

The Quality Index [1] is a systematic checklist designed to evaluate the reporting quality, internal validity and external validity of clinical research articles. When applied to non-randomised studies, the quality index has been reported to have good test-retest ($r = 0.79$) and inter-rater ($r = 0.77$) reliability [1]. The index was adjusted to exclude 12 questions that could not be applied to observational studies with a case-control design, resulting in the retention of 15 questions (Supplementary Table 2). One question on the reporting of statistical values (Q10) was obtained from an alternative version of the Quality Index, as described by MacLehose et al [2].

Supplementary Table 2. Questions included from the Quality Index

**1**	Is the objective of the study clearly described ?
**2**	Are the main outcomes to be measured clearly described in the Introduction or Methods section?
**3**	Are the characteristics of the patients included in the study clearly described?
**5**	Are the distributions of principal confounders in each group of subjects to be compared clearly described?
**6**	Are the main findings of the study clearly described?
**7**	Does the study provide estimates of the random variability in the data for the main outcomes?
**10**	Have 95% CI's and/or actual *p* values been reported for the main outcomes, except where the *p* value is less than 0.001?
**12**	Were those subjects who were prepared to participate representative of the entire population from which they were recruited?
**15**	Was an attempt made to blind those measuring the main outcomes of the intervention?
**16**	If any of the results of the study were based on "data dredging", was this made clear?
**18**	Were the statistical tests used to assess the main outcomes appropriate?
**20**	Were the main outcome measures used accurate (valid and reliable)?
**21**	Were the cases and controls recruited from the same population?
**22**	Were cases and controls recruited over the same period of time?
**25**	Was there adequate adjustment for confounding in the analyses from which the main findings were drawn?

Additional Data File 2. References

1. Downs SH, Black N: **The feasibility of creating a checklist for the assessment of the methodological quality both of randomised and non-randomised studies of health care interventions**. *J Epidemiol Community Health* 1998, **52**(6):377-384.
2. MacLehose RR, Reeves BC, Harvey IM, Sheldon TA, Russell IT, Black AM: **A systematic review of comparisons of effect sizes derived from randomised and non-randomised studies**. *Health Technol Assess* 2000, **4**(34):1-154.
