# Supplementary material for: Diagnostic imaging for chronic plantar heel pain: a systematic review and meta-analysis
Source: J Foot Ankle Res. 2009 Nov 13;2:32. doi: 10.1186/1757-1146-2-32 (PMC2784446; doi:10.1186/1757-1146-2-32)
Supplement: Additional file 3 — Search results by database. A table showing the number of citations generated by the search strategy for each database. [file 1757-1146-2-32-S3.pdf]

Diagnostic imaging for chronic plantar heel pain: a systematic review and meta-analysis

Andrew M. McMillan, Karl B. Landorf, Joanna T. Barrett, Hylton B. Menz, Adam R. Bird

Additional Data File 3. Search results by database.

**Medline**	422
**EMBASE**	431
**CINAHL**	196
**Sport Discus**	134
**Cochrane Library**	1
**Total**	1184
**Total without duplication**	764
