# Supplementary material for: Diagnostic imaging for chronic plantar heel pain: a systematic review and meta-analysis
Source: J Foot Ankle Res. 2009 Nov 13;2:32. doi: 10.1186/1757-1146-2-32 (PMC2784446; doi:10.1186/1757-1146-2-32)
Supplement: Additional file 4 — Included studies. A table showing the author and publication details of the included studies. [file 1757-1146-2-32-S4.pdf]

Diagnostic imaging for chronic plantar heel pain: a systematic review and meta-analysis

Andrew M. McMillan, Karl B. Landorf, Joanna T. Barrett, Hylton B. Menz, Adam R. Bird

Additional Data File 4. Included studies

Principal Author	Year	Title	Journal
Akfirat	2003	Ultrasonographic appearance of the plantar fasciitis.	Clin Imaging
Berkowitz	1991	Plantar fasciitis: MR imaging.	Radiology
Bygrave	1998	Diagnosing plantar fasciitis with ultrasound using Planscan.	Foot
Cardinal	1996	Plantar fasciitis: sonographic evaluation.	Radiology
Cetin	2001	Evaluation of chronic plantar fasciitis by scintigraphy and relation to clinical parameters.	J Musculoskeletal Pain
Genc	2005	Long-term ultrasonographic follow-up of plantar fasciitis patients treated with steroid injection.	Joint Bone Spine
Gibbon	1999	Ultrasound of the plantar aponeurosis (fascia).	Skeletal Radiol
Hall	1996	Magnetic resonance imaging in the evaluation of heel pain.	Orthopedics
Kamel	2000	High frequency ultrasonographic findings in plantar fasciitis and assessment of local steroid injection.	J Rheumatol
Karabay	2007	Ultrasonographic evaluation in plantar fasciitis.	J Foot Ankle Surg
O'Duffy	1998	Foot pain: specific indications for scintigraphy.	Br J Rheumatol
Osborne	2006	Critical differences in lateral X-rays with and without a diagnosis of platar fasciitis.	J Sci & Med in Sport
Ozdemir	2005	Sonographic evaluation of plantar fasciitis and relation to body mass index.	Eur J Radiol
Prichasuk	1994	The relationship of pes planus and calcaneal spur to plantar heel pain.	Clin Orthop
Sabir	2005	Clinical utility of sonography in diagnosing plantar fasciitis.	J Ultrasound Med
Tsai	2000	Ultrasound evaluation of plantar fasciitis.	Scand J Rheumatol
Turgut	1999	The relationship of heel pad elasticity and plantar heel pain.	Clin Orthop
Vohra	2002	Ultrasonographic evaluation of plantar fascia bands. A retrospective study of 211 symptomatic feet.	J Am Podiatr Med Assoc
Wainwright	1995	Calcaneal spurs and plantar fasciitis.	Foot
Wall	1993	Ultrasound diagnosis of plantar fasciitis.	Foot Ankle
Walther	2004	Power Doppler findings in plantar fasciitis.	Ultrasound Med Biol
Wearing	2007	Plantar fasciitis: are pain and fascial thickness associated with arch shape and loading?	Phys Ther
Williams	1987	Imaging study of the painful heel syndrome.	Foot Ankle
