# Supplementary material for: Diagnostic imaging for chronic plantar heel pain: a systematic review and meta-analysis
Source: J Foot Ankle Res. 2009 Nov 13;2:32. doi: 10.1186/1757-1146-2-32 (PMC2784446; doi:10.1186/1757-1146-2-32)
Supplement: Additional file 5 — Exclusion grounds for articles rejected after full-text assessment. A table showing the exclusion grounds for articles excluded from the review after full-text assessment. [file 1757-1146-2-32-S5.pdf]

## Diagnostic imaging for chronic plantar heel pain: a systematic review and meta-analysis

Andrew M. McMillan, Karl B. Landorf, Joanna T. Barrett, Hylton B. Menz, Adam R. Bird

### Additional Data File 5. Exclusion grounds for articles rejected after full-text assessment

| | Principal Author | Year | Grounds for Exclusion |
|---|---|---|---|
| 1 | Breunung | 2008 | Single case report |
| 2 | Buchbinder | 2002 | No imaging comparison to independent control group |
| 3 | Chigwanda | 1997 | No imaging comparison to independent control group |
| 4 | Chundru | 2008 | Comparison of patients with and without muscular atrophy |
| 5 | Cosentino | 2001 | No imaging comparison to independent control group |
| 6 | Cosentino | 2004 | No imaging comparison to independent control group |
| 7 | Falsetti | 2003 | Disease specific cohort |
| 8 | Frater | 2006 | No imaging comparison to independent control group |
| 9 | Grasel | 1999 | No imaging comparison to independent control group |
| 10 | Groshar | 2000 | Comparison to asymptomatic foot only |
| 11 | Hammer | 2005 | Comparison to asymptomatic foot only |
| 12 | Heim | 2000 | Case reports |
| 13 | Howells | 1994 | Letter and author reply |
| 14 | Intenzo | 1991 | No imaging comparison to independent control group |
| 15 | Kamel | 2003 | Disease specific cohort: seronegative arthropathy |
| 16 | Kane | 1998 | Comparison to asymptomatic foot only |
| 17 | Kane | 2001 | Comparison to asymptomatic foot only |
| 18 | Kell | 1994 | Case reports |
| 19 | Kier | 1991 | No comparative research, graphical descriptions only |
| 20 | Koulouris | 2005 | No comparative research |
| 21 | Levy | 2006 | No imaging comparison to independent control group |
| 22 | Liang | 2007 | No imaging comparison to independent control group |
| 23 | Maier | 2000 | No imaging comparison to independent control group |
| 24 | McGonagle | 2002 | No imaging comparison to independent control group |
| 25 | Ozdemir | 2002 | No imaging comparison to independent control group |
| 26 | Rapp | 2006 | No imaging comparison to independent control group, not published in a peer-reviewed journal |
| 27 | Reinherz | 1989 | No research findings presented |
| 28 | Roger | 1997 | Pictorial review only |
| 29 | Sewell | 1980 | No imaging comparison to independent control group |
| 30 | Sorrentino | 2008 | No imaging comparison to independent control group |
| 31 | Tanz | 1963 | Various causes of plantar heel pain, including trauma |
| 32 | Theodorou | 2000 | No imaging comparison to independent control group |
| 33 | Theodorou | 2002 | No imaging comparison to independent control group |
| 34 | Tsai | 2000 | No imaging comparison to independent control group |
| 35 | Tudor | 1997 | No imaging comparison to independent control group |
| 36 | Uzel | 2006 | No imaging comparison to independent control group |
| 37 | Wearing | 2004 | Unable to verify if imaging results had been published elsewhere |
