# Supplementary material for: Diagnostic imaging for chronic plantar heel pain: a systematic review and meta-analysis
Source: J Foot Ankle Res. 2009 Nov 13;2:32. doi: 10.1186/1757-1146-2-32 (PMC2784446; doi:10.1186/1757-1146-2-32)
Supplement: Additional file 6 — Quality Index Scores. A table showing the individual quality index scores for each included article. [file 1757-1146-2-32-S6.pdf]

# Diagnostic imaging for chronic plantar heel pain: a systematic review and meta-analysis

Andrew M. McMillan, Karl B. Landorf, Joanna T. Barrett, Hylton B. Menz, Adam R. Bird

## Additional Data File 6. Quality Index Scores

| | Question | | | | | | | | | | | | | | | |
|---|---|---|---|---|---|---|---|---|---|---|---|---|---|---|---|---|
| | 1 | 2 | 3 | 5 | 6 | 7 | 10 | 12 | 15 | 16 | 18 | 20 | 21 | 22 | 25 | % |
| **Akfirat [1]** | 1 | 0 | 1 | 1 | 1 | 1 | 0 | 0 | 0 | 1 | 1 | 0 | 0 | 0 | 1 | **53** |
| **Berkowitz [2]** | 1 | 1 | 0 | 0 | 1 | 1 | 0.5 | 0 | 0 | 1 | 0 | 1 | 0 | 0 | 0 | **43** |
| **Bygrave [3]** | 1 | 1 | 0 | 1 | 1 | 1 | 0.5 | 0 | 0 | 1 | 1 | 1 | 0 | 0 | 0 | **57** |
| **Cardinal [4]** | 1 | 1 | 0 | 0 | 1 | 1 | 0.5 | 0 | 0 | 1 | 0 | 0 | 0 | 0 | 0 | **37** |
| **Cetin [5]** | 1 | 1 | 0 | 1 | 1 | n/a | 0 | 0 | 0 | 1 | 0 | 0 | 1 | 0 | 1 | **50** |
| **Genc [6]** | 1 | 1 | 0 | 1 | 1 | 1 | 0.5 | 0 | 1 | 1 | 1 | 1 | 1 | 0 | 1 | **77** |
| **Gibbon [7]** | 1 | 1 | 0 | 0 | 1 | 0 | 0.5 | 0 | 0 | 1 | 1 | 1 | 0 | 0 | 0 | **43** |
| **Hall [8]** | 1 | 0 | 1 | 0 | 0 | n/a | 0 | 0 | 0 | 1 | 0 | 0 | 1 | 0 | 0 | **29** |
| **Kamel [9]** | 1 | 1 | 0 | 0 | 1 | 1 | 0.5 | 0 | 0 | 1 | 0 | 1 | 0 | 0 | 0 | **43** |
| **Karabay [10]** | 0 | 1 | 0 | 0 | 1 | 1 | 0 | 0 | 0 | 1 | 0 | 1 | 0 | 0 | 1 | **40** |
| **O'Duffy [11]** | 1 | 1 | 1 | 0 | 1 | n/a | 0 | 0 | 0 | 1 | 0 | 0 | 1 | 0 | 1 | **50** |
| **Osborne [12]** | 1 | 1 | 1 | 0 | 1 | n/a | 0.5 | 0 | 1 | 1 | 1 | 1 | 0 | 0 | 1 | **68** |
| **Ozdemir [13]** | 1 | 1 | 0 | 1 | 1 | 1 | 0 | 1 | 1 | 1 | 1 | 1 | 0 | 0 | 0 | **67** |
| **Prichasuk [14]** | 1 | 1 | 1 | 0 | 1 | n/a | 0.5 | 0 | 0 | 1 | 1 | 1 | 0 | 1 | 0 | **61** |
| **Sabir [15]** | 1 | 1 | 0 | 1 | 1 | 1 | 0.5 | 0 | 1 | 1 | 1 | 1 | 0 | 1 | 0 | **70** |
| **Tsai [16]** | 1 | 1 | 0 | 1 | 1 | 1 | 0.5 | 1 | 1 | 1 | 1 | 1 | 0 | 0 | 1 | **77** |
| **Turgut [17]** | 1 | 1 | 1 | 0 | 1 | 1 | 0 | 0 | 1 | 1 | 1 | 1 | 1 | 1 | 1 | **80** |
| **Vohra [18]** | 1 | 1 | 1 | 0 | 1 | 0 | 0.5 | 0 | 0 | 1 | 1 | 0 | 0 | 0 | 1 | **50** |
| **Wainwright [19]** | 1 | 1 | 1 | 0 | 1 | n/a | 0.5 | 0 | 0 | 1 | 1 | 1 | 1 | 0 | 0 | **61** |
| **Wall [20]** | 1 | 1 | 0 | 1 | 1 | 1 | 1 | 0 | 1 | 1 | 1 | 1 | 0 | 0 | 1 | **73** |
| **Walther [21]** | 1 | 1 | 0 | 0 | 1 | 1 | 0 | 0 | 0 | 1 | 1 | 0 | 0 | 0 | 1 | **47** |
| **Wearing [22]** | 1 | 1 | 0 | 1 | 1 | 1 | 0 | 0 | 0 | 1 | 0 | 1 | 0 | 0 | 1 | **53** |
| **Williams [23]** | 0 | 0 | 1 | 0 | 1 | n/a | 0 | 0 | 1 | 1 | 0 | 0 | 1 | 0 | 0 | **36** |

**Additional Data File 6. References**


1. Akfirat M, Sen C, Gunes T: **Ultrasonographic appearance of the plantar fasciitis**. *Clin Imaging* 2003, **27**(5):353-357.
2. Berkowitz JF, Kier R, Rudicel S: **Plantar fasciitis: M.R imaging**. *Radiology* 1991, **179**(3):665-667.
3. Bygrave CJ, Betts RP, Saxelby J: **Diagnosing plantar fasciitis with ultrasound using Planscan**. *Foot* 1998, **8**(3):141-146.
4. Cardinal E, Chhem RK, Beauregard CG, Aubin B, Pelletier M: **Plantar fasciitis: sonographic evaluation.** *Radiology* 1996, **201**(1):257-259.
5. Cetin A, Sivri A, Dincer F, Kiratli P, Ceylan E: **Evaluation of chronic plantar fasciitis by scintigraphy and relation to clinical parameters**. *J Musculoskeletal Pain* 2001, **9**(4):55-61.
6. Genc H, Saracoglu M, Nacir B, Erdem HR, Kacar M: **Long-term ultrasonographic follow-up of plantar fasciitis patients treated with steroid injection**. *Joint Bone Spine* 2005, **72**(1):61-65.
7. Gibbon WW, Long G: **Ultrasound of the plantar aponeurosis (fascia)**. *Skeletal Radiol* 1999, **28**(1):21-26.
8. Hall RL, Erickson SJ, Shereff MJ, Johnson JE, Kneeland JB: **Magnetic resonance imaging in the evaluation of heel pain**. *Orthopedics* 1996, **19**(3):225-229.
9. Kamel M, Kotob H: **High frequency ultrasonographic findings in plantar fasciitis and assessment of local steroid injection**. *J Rheumatol* 2000, **27**(9):2139-2141.
10. Karabay N, Toros T, Hurel C: **Ultrasonographic evaluation in plantar fasciitis**. *J Foot Ankle Surg* 2007, **46**(6):442-446.
11. O'Duffy EK, Clunie GP, Gacinovic S, Edwards JC, Bomanji JB, Ell PJ: **Foot pain: specific indications for scintigraphy**. *Br J Rheumatol* 1998, **37**(4):442-447.
12. Osborne HR, Breidahl WH, Allison GT: **Critical differences in lateral X-rays with and without a diagnosis of platar fasciitis**. *J Sci Med Sport* 2006, **9**(3):231-237.
13. Ozdemir H, Yilmaz E, Murat A, Karakurt L, Poyraz AK, Ogur E: **Sonographic evaluation of plantar fasciitis and relation to body mass index**. *Eur J Radiol* 2005, **54**(3):443-447.
14. Prichasuk S, Subhadrabandhu T: **The relationship of pes planus and calcaneal spur to plantar heel pain.** *Clin Orthop* 1994(306):192-196.
15. Sabir N, Demirlenk S, Yagci B, Karabulut N, Cubukcu S: **Clinical utility of sonography in diagnosing plantar fasciitis**. *J Ultrasound Med* 2005, **24**(8):1041-1048.
16. Tsai WC, Chiu MF, Wang CL, Tang FT, Wong MK: **Ultrasound evaluation of plantar fasciitis.** *Scand J Rheumatol* 2000, **29**(4):255-259.
17. Turgut A, Gokturk E, Kose N, Seber S, Hazer B, Gunal I: **The relationship of heel pad elasticity and plantar heel pain**. *Clin Orthop* 1999(360):191-196.
18. Vohra PK, Kincaid BR, Japour CJ, Sobel E: **Ultrasonographic evaluation of plantar fascia bands. A retrospective study of 211 symptomatic feet**. *J Am Podiatr Med Assoc* 2002, **92**(8):444-449.
19. Wainwright AM, Kelly AJ, Winson IG: **Calcaneal spurs and plantar fasciitis**. *Foot* 1995, **5**(3):123-126.

20. Wall JR, Harkness MA, Crawford A: **Ultrasound diagnosis of plantar fasciitis**. *Foot Ankle* 1993, **14**(8):465-470.
21. Walther M, Radke S, Kirschner S, Ettl V, Gohlke F: **Power Doppler findings in plantar fasciitis**. *Ultrasound Med Biol* 2004, **30**(4):435-440.
22. Wearing SC, Smeathers JE, Sullivan PM, Yates B, Urry SR, Dubois P: **Plantar fasciitis: are pain and fascial thickness associated with arch shape and loading?** *Phys Ther* 2007, **87**(8):1002-1008.
23. Williams PL, Smibert JG, Cox R, Mitchell R, Klenerman L: **Imaging study of the painful heel syndrome**. *Foot Ankle* 1987, **7**(6):345-349.
