# Supplementary material for: Diagnostic imaging for chronic plantar heel pain: a systematic review and meta-analysis
Source: J Foot Ankle Res. 2009 Nov 13;2:32. doi: 10.1186/1757-1146-2-32 (PMC2784446; doi:10.1186/1757-1146-2-32)
Supplement: Additional file 7 — Thickness of the proximal plantar fascia by ultrasonography: variability between studies. A detailed description of the methodological variability between studies reporting plantar fascia thickness by ultrasonography. [file 1757-1146-2-32-S7.pdf]

Diagnostic imaging for chronic plantar heel pain: a systematic review and meta-analysis

Andrew M. McMillan, Karl B. Landorf, Joanna T. Barrett, Hylton B. Menz, Adam R. Bird

---

Additional Data File 7. Thickness of the proximal plantar fascia by ultrasonography: variability between studies

Condition groups ranged in size from 10 to 109 participants, and with the exception of two groups [1, 2] all included more females than males. The mean age of condition participants was not reported in three studies [1, 3, 4], and ranged from 43 to 53 years for the remaining 10 groups. The mean BMI of condition participants was reported in five studies [1, 5-8], ranging from 24.5 to 34.2. All studies used the term 'plantar fasciitis' to describe the diagnosis of condition participants, one study [2] also included 'inferior calcaneal spur syndrome' and another study [9] 'plantar heel pain'. Seven studies [1, 4, 5, 8-11] reported the clinical features of condition participants, all of which described localised pain at either the medial calcaneal tubercle or plantar fascia origin. The mean duration of symptoms was reported in three studies [5, 8, 9], ranging from 9 to 12 months.

Control groups ranged in size from 10 to 77 participants, and with the exception of three groups [4, 11, 12] all included more females than males. The sex distribution in one control group was not reported [10]. The mean age of control participants was not reported in four studies [1,3,4,10], and ranged from 36 to 49 years for the remaining nine groups. The mean BMI of control participants was reported in five studies [1, 5-8], ranging from 23.3 to 28.3.

Participants from nine studies [2, 3, 6-11, 13] were positioned prone with their knees extended and feet hanging over the edge of the examination table. Three studies [4, 5, 12] did not report participant position, and participants from one study [1] were seated. All studies measured plantar fascia thickness in a sagittal view with linear array transducers ranging from 5 to 12 MHz. Measurements were taken in a very similar location across all studies: four studies [1, 6, 9, 12] measured fascia thickness at 5mm distal to either the calcaneal insertion or medial calcaneal tubercle, five studies [4,8,10,11,13] near the calcaneal insertion, and four studies [2,3,5,7] at the anterior edge of the inferior calcaneal border.

Additional Data File 7. References

1. Bygrave CJ, Betts RP, Saxelby J: **Diagnosing plantar fasciitis with ultrasound using Planscan**. *Foot* 1998, **8**(3):141-146.
2. Gibbon WW, Long G: **Ultrasound of the plantar aponeurosis (fascia)**. *Skeletal Radiol* 1999, **28**(1):21-26.
3. Kamel M, Kotob H: **High frequency ultrasonographic findings in plantar fasciitis and assessment of local steroid injection**. *J Rheumatol* 2000, **27**(9):2139-2141.
4. Karabay N, Toros T, Hurel C: **Ultrasonographic evaluation in plantar fasciitis**. *J Foot Ankle Surg* 2007, **46**(6):442-446.
5. Genc H, Saracoglu M, Nacir B, Erdem HR, Kacar M: **Long-term ultrasonographic follow-up of plantar fasciitis patients treated with steroid injection**. *Joint Bone Spine* 2005, **72**(1):61-65.
6. Ozdemir H, Yilmaz E, Murat A, Karakurt L, Poyraz AK, Ogur E: **Sonographic evaluation of plantar fasciitis and relation to body mass index**. *Eur J Radiol* 2005, **54**(3):443-447.
7. Sabir N, Demirlenk S, Yagci B, Karabulut N, Cubukcu S: **Clinical utility of sonography in diagnosing plantar fasciitis**. *J Ultrasound Med* 2005, **24**(8):1041-1048.
8. Tsai WC, Chiu MF, Wang CL, Tang FT, Wong MK: **Ultrasound evaluation of plantar fasciitis.** *Scand J Rheumatol* 2000, **29**(4):255-259.
9. Wearing SC, Smeathers JE, Sullivan PM, Yates B, Urry SR, Dubois P: **Plantar fasciitis: are pain and fascial thickness associated with arch shape and loading?** *Phys Ther* 2007, **87**(8):1002-1008.
10. Cardinal E, Chhem RK, Beauregard CG, Aubin B, Pelletier M: **Plantar fasciitis: sonographic evaluation.** *Radiology* 1996, **201**(1):257-259.
11. Vohra PK, Kincaid BR, Japour CJ, Sobel E: **Ultrasonographic evaluation of plantar fascia bands. A retrospective study of 211 symptomatic feet**. *J Am Podiatr Med Assoc* 2002, **92**(8):444-449.
12. Wall JR, Harkness MA, Crawford A: **Ultrasound diagnosis of plantar fasciitis**. *Foot Ankle* 1993, **14**(8):465-470.
13. Walther M, Radke S, Kirschner S, Ettl V, Gohlke F: **Power Doppler findings in plantar fasciitis**. *Ultrasound Med Biol* 2004, **30**(4):435-440.
