# Supplementary material for: Diagnostic imaging for chronic plantar heel pain: a systematic review and meta-analysis
Source: J Foot Ankle Res. 2009 Nov 13;2:32. doi: 10.1186/1757-1146-2-32 (PMC2784446; doi:10.1186/1757-1146-2-32)
Supplement: Additional file 8 — Thickness of the plantar fascia by MRI: variability between studies. A detailed description of the methodological variability between studies reporting plantar fascia thickness by MRI. [file 1757-1146-2-32-S8.pdf]

Diagnostic imaging for chronic plantar heel pain: a systematic review and meta-analysis

Andrew M. McMillan, Karl B. Landorf, Joanna T. Barrett, Hylton B. Menz, Adam R. Bird

---

Additional Data File 8. Thickness of the plantar fascia by MRI: variability between studies

The size of condition groups were 77 participants [1] and 8 participants [2], with both groups including more females than males. The mean age of condition participants was very similar at 45 and 43 years. One study [1] reported the mean BMI of condition participants, with a value of 34.2. Both studies used the term ‘plantar fasciitis’ to describe the diagnosis of condition participants, though neither study reported the mean duration of symptoms.

The size of control groups were 77 participants [1] and 5 participants [2], with one group including more females than males [1] and the other only females [2]. The mean age of control participants was also very similar at 42 and 41 years. One study [1] reported the mean BMI of control participants, with a value of 25.

Both studies used a 1.5-T MRI unit with a superconductive system and extremity coil. Sagittal T1-weighted sequences were obtained in both studies with similar repetition (400-600 milliseconds) and echo times (18-20 milliseconds). Measurements of plantar fascia thickness were taken near the calcaneal insertion in both studies.

Additional Data File 8. References

1. Sabir N, Demirlenk S, Yagci B, Karabulut N, Cubukcu S: **Clinical utility of sonography in diagnosing plantar fasciitis**. *J Ultrasound Med* 2005, **24**(8):1041-1048.
2. Berkowitz JF, Kier R, Rudicel S: **Plantar fasciitis: M.R imaging**. *Radiology* 1991, **179**(3):665-667.
