# Supplementary material for: Diagnostic imaging for chronic plantar heel pain: a systematic review and meta-analysis
Source: J Foot Ankle Res. 2009 Nov 13;2:32. doi: 10.1186/1757-1146-2-32 (PMC2784446; doi:10.1186/1757-1146-2-32)
Supplement: Additional file 9 — Echogenicity of the proximal plantar fascia: variability between studies. A detailed description of the methodological variability between studies reporting plantar fascia echogenicity. [file 1757-1146-2-32-S9.pdf]

Diagnostic imaging for chronic plantar heel pain: a systematic review and meta-analysis

Andrew M. McMillan, Karl B. Landorf, Joanna T. Barrett, Hylton B. Menz, Adam R. Bird

Additional Data File 9. Echogenicity of the proximal plantar fascia: variability between studies

Condition groups ranged in size from 15 to 102 participants and included more females than males. The mean age of condition participants ranged from 43 to 45 years. The mean BMI of condition participants was reported in two studies [1, 2] with values of 24.5 and 28.1. All studies used the term ‘plantar fasciitis’ to describe the diagnosis of condition participants. Three studies reported the clinical features of condition participants [1-3], all of which described heel pain localised to either the medial calcaneal tubercle or plantar fascia origin. Two studies [1, 2] reported the mean duration of symptoms, with values of 12 and 11 months.

The four control groups ranged in size from 15 to 33 participants. Three studies reported the mean age of control participants [1, 2, 4], ranging from 41 to 42 years. The same three studies reported the sex distribution in control groups, all of which included more females than males. The mean BMI of control participants was reported in two studies [1, 2], with values of 28.3 and 23.3.

The position of participants during examination was reported in three studies [2-4], with participants placed prone with knees extended. All studies observed the plantar fascia in a sagittal view with linear array transducers ranging from 7 to 12 MHz, and reported qualitative changes in echogenicity near the calcaneal insertion.

Additional Data File 9. References

1. Genc H, Saracoglu M, Nacir B, Erdem HR, Kacar M: **Long-term ultrasonographic follow-up of plantar fasciitis patients treated with steroid injection**. *Joint Bone Spine*2005, **72**(1):61-65.
2. Tsai WC, Chiu MF, Wang CL, Tang FT, Wong MK: **Ultrasound evaluation of plantar fasciitis.** *Scand J Rheumatol* 2000, **29**(4):255-259.
3. Cardinal E, Chhem RK, Beauregard CG, Aubin B, Pelletier M: **Plantar fasciitis: sonographic evaluation.** *Radiology* 1996, **201**(1):257-259.
4. Walther M, Radke S, Kirschner S, Ettl V, Gohlke F: **Power Doppler findings in plantar fasciitis**. *Ultrasound Med Biol* 2004, **30**(4):435-440.
