# Supplementary material for: Diagnostic imaging for chronic plantar heel pain: a systematic review and meta-analysis
Source: J Foot Ankle Res. 2009 Nov 13;2:32. doi: 10.1186/1757-1146-2-32 (PMC2784446; doi:10.1186/1757-1146-2-32)
Supplement: Additional file 10 — Evidence of plantar calcaneal spur by plain film x-ray: variability between studies. A detailed description of the methodological variability between studies reporting evidence of plantar calcaneal spur by plain film x-ray. [file 1757-1146-2-32-S10.pdf]

Diagnostic imaging for chronic plantar heel pain: a systematic review and meta-analysis

Andrew M. McMillan, Karl B. Landorf, Joanna T. Barrett, Hylton B. Menz, Adam R. Bird

Additional Data File 10. Evidence of plantar calcaneal spur by plain film x-ray: variability between studies

Condition groups ranged in size from 21 to 82 participants, and with the exception of two groups [1, 2] all included more females than males. The mean age of condition participants ranged from 45 to 55 years. The mean BMI of condition participants was reported in four studies [2-5], ranging from 26.3 to 29.2. Four studies used the term 'plantar fasciitis' to describe the diagnosis of condition participants, two studies used the term 'plantar heel pain' and one study 'painful heel syndrome'. Five studies reported the clinical features of condition participants, all of which described localised pain either beneath the heel, at the medial calcaneal tubercle or plantar fascia origin. The mean duration of symptoms was reported in two studies [2, 4] with values of 5.7 and 6.78 months.

Control groups ranged from 15 to 400 participants with more females in three groups [3, 4, 6], more males in two groups [1,2], and an equal proportion in one group [5]. The sex distribution in one study [7] was not reported. Four studies reported the mean age of control participants [1, 3, 4, 6], ranging from 43 to 53 years. The mean BMI of control participants was reported in three studies [3-5], with values from 22.6 to 28.7.

X-ray projection was not reported in one study [7], however the remaining six all obtained plain lateral radiographs for assessment. The presence of subcalcaneal spur was a subjective observation for all except one study [5] in which objective criteria was applied (>2mm horizontal projection). Consensus between two independent observers occurred in two studies [2,6].

Additional Data File 10. References

1. Osborne HR, Breidahl WH, Allison GT: **Critical differences in lateral X-rays with and without a diagnosis of platar fasciitis**. *J Sci Med Sport* 2006, **9**(3):231-237.
2. Williams PL, Smibert JG, Cox R, Mitchell R, Klenerman L: **Imaging study of the painful heel syndrome**. *Foot Ankle* 1987, **7**(6):345-349.
3. Akfirat M, Sen C, Gunes T: **Ultrasonographic appearance of the plantar fasciitis**. *Clin Imaging* 2003, **27**(5):353-357.
4. Cetin A, Sivri A, Dincer F, Kiratli P, Ceylan E: **Evaluation of chronic plantar fasciitis by scintigraphy and relation to clinical parameters**. *J Musculoskeletal Pain* 2001, **9**(4):55-61.
5. Prichasuk S, Subhadrabandhu T: **The relationship of pes planus and calcaneal spur to plantar heel pain.** *Clin Orthop* 1994(306):192-196.
6. Wainwright AM, Kelly AJ, Winson IG: **Calcaneal spurs and plantar fasciitis**. *Foot* 1995, **5**(3):123-126.
7. Turgut A, Gokturk E, Kose N, Seber S, Hazer B, Gunal I: **The relationship of heel pad elasticity and plantar heel pain**. *Clin Orthop* 1999(360):191-196.
